# Localized primary gastrointestinal diffuse large B cell lymphoma received a surgical approach: an analysis of prognostic factors and comparison of staging systems in 101 patients from a single institution

**DOI:** 10.1186/s12957-015-0668-5

**Published:** 2015-08-15

**Authors:** Shengting Zhang, Li Wang, Dong Yu, Yang Shen, Shu Cheng, Li Zhang, Ying Qian, Zhixiang Shen, Qinyu Li, Weili Zhao

**Affiliations:** State Key Laboratory of Medical Genomics, Shanghai Institute of Hematology, Shanghai Rui Jin Hospital, Shanghai Jiao Tong University School of Medicine, Shanghai, China; Department of Surgery, Shanghai Rui Jin Hospital, Shanghai Jiao Tong University School of Medicine, 197 Rui Jin Er Road, Shanghai, 200025 China

**Keywords:** Primary gastrointestinal lymphoma, DLBCL, Prognosis, Surgical approach, Prognostic staging system

## Abstract

**Background:**

Diffuse large B cell lymphoma (DLBCL) represents the most common histological subtype of primary gastrointestinal lymphoma and is a heterogeneous group of disease. Prognostic characterization of individual patients is an essential prerequisite for a proper risk-based therapeutic choice.

**Methods:**

Clinical and pathological prognostic factors were identified, and predictive value of four previously described prognostic systems were assessed in 101 primary gastrointestinal DLBCL (PG-DLBCL) patients with localized disease, including Ann Arbor staging with Musshoff modification, International Prognostic Index (IPI), Lugano classification, and Paris staging system.

**Results:**

Univariate factors correlated with inferior survival time were clinical parameters [age >60 years old, multiple extranodal/gastrointestinal involvement, elevated serum lactate dehydrogenase and β2-microglobulin, and decreased serum albumin], as well as pathological parameters (invasion depth beyond serosa, involvement of regional lymph node or adjacent tissue, Ki-67 index, and Bcl-2 expression). Major independent variables of adverse outcome indicated by multivariate analysis were multiple gastrointestinal involvement. In patients unfit for Rituximab but received surgery, radical surgery significantly prolonged the survival time, comparing with alleviative surgery. Addition of Rituximab could overcome the negative prognostic effect of alleviative surgery. Among the four prognostic systems, IPI and Lugano classification clearly separated patients into different risk groups. IPI was able to further stratify the early-stage patients of Lugano classification into groups with distinct prognosis.

**Conclusions:**

Radical surgery might be proposed for the patients unfit for Rituximab treatment, and a combination of clinical and pathological staging systems was more helpful to predict the disease outcome of PG-DLBCL patients.

**Electronic supplementary material:**

The online version of this article (doi:10.1186/s12957-015-0668-5) contains supplementary material, which is available to authorized users.

## Background

Non-Hodgkin’s lymphoma of the gastrointestinal tract is the most common extranodal lymphoma, accounting for 30–40 % of the patients, in which diffuse large B cell lymphoma (DLBCL) is the most frequent histological subtype with a variable clinical outcome [1, 2]. Therefore, prognostic characterization of individual patients is an essential prerequisite for a proper risk-based therapeutic choice.

Surgery was once the standard procedure or a regular component of combined treatment modalities in primary gastrointestinal DLBCL (PG-DLBCL) [3]. The factors in favor of a surgical approach include the removal of primary lesions, availability of precise histological classification and staging, as well as avoidance of complications such as perforation or hemorrhage that may occur during radiotherapy and chemotherapy [4–6]. In the recent years, opinion has increasingly swung toward non-invasive treatment even for patients with resectable disease, so as to maintain their quality of life [7–9]. However, the benefit of a surgical approach remains controversial in the patients treated with Rituximab and chemotherapy/radiotherapy.

Several staging systems have been developed over the past decades to improve prognostic stratification of primary gastrointestinal lymphoma, mainly taking into account different clinical parameters. The classical Ann Arbor staging system is adapted for extranodal lymphoma, as proposed by Musshoff et al. [10]. International Prognostic Index (IPI) is originated from patients with DLBCL that consists of age, performance status, Ann Arbor stage, serum lactate dehydrogenase (LDH), and extranodal involvement [11]. Meanwhile, novel scores have been explored, namely Lugano classification and Paris staging system [12, 13], which combine clinical features with pathological findings of the tumors.

Although both clinical and pathological prognostic systems were effective in the patient series used to drive them, their utility needs testing in other patient populations of PG-DLBCL, particularly in those with accurate pathological data obtained by a surgical approach. Moreover, direct evaluation and comparison of these systems are limited in large -scale Chinese patients with localized disease in the Rituximab era. To address this issue, we conducted a retrospective analysis of 101 patients followed up in our Institution over the last 12 years to identify the main prognostic factors and to compare different staging systems in the prediction of survival in localized PG-DLBCL that received a surgical approach.

## Methods

### Patients

From January 2003 to October 2014, a total of 101 patients of localized PG-DLBCL received a surgical approach were included in this retrospective study and 49 patients who received chemotherapy alone were referred as control. PG-DLBCL was defined according to Lewin et al.: patients had to present gastrointestinal symptoms or predominant lesions in the gastrointestinal tract [14]. Informed consent was obtained from all patients, in accordance with the regulations of the Shanghai Jiao Tong University School of Medicine Institutional Review Boards.

### Diagnosis and staging systems

Pathological diagnosis was established according to the World Health Organization (WHO) classification [15]. The staging work-up included history and physical examination, blood cell counts and serum chemistry, bone marrow aspiration or biopsy, endoscopy of gastrointestinal tracts, and chest and abdominal tomography scan or positron emission tomography-computerized tomography (PET-CT). The stage of lymphoma was assessed following the guidelines of Ann Arbor staging with Musshoff modification (Ie_1_/Ie_2_/IIe_1_/IIe_2_), IPI (low/low–intermediate/high–intermediate/high-risk), Lugano classification (I/II_1_/II_2_/IIE), and Paris staging system (TxNxMxBx), respectively. The macroscopic type of lymphoma (ulcerative, diffuse, or massive type), the depth of tumor invasion, as well as the involvement of regional lymph nodes and adjacent structure were determined based on histology of the resected tumor specimens.

### Treatment and response

A standard radical gastrectomy is defined as a gastrectomy with D2 lymphadenectomy and resection of Nl and N2 lymph nodes [16]. Radical surgery for primary intestinal lymphoma is defined as completely primary mass resection and regional lymph nodes dissection. As for the alleviative surgery, the lesions were not completely resected, which is also called surgical debulking, including local mass resection (R1–R2), enterostomy, and simple perforation repair [17, 18].

The patients received a surgical approach, either alone or followed by chemotherapy (four to six standard dose of CHOP regimens) or combined with Rituximab (375 mg/m^2^). The treatment response was evaluated according to the WHO response criteria. Complete response (CR) was defined as no evidence of residual disease, partial response (PR) with at least 50 % reduction in tumor burden from the onset of treatment, and stable disease (SD) and progression disease (PD) with less than 50 % reduction in tumor burden or disease progression. Assessment of treatment response was evaluated by clinical follow-up, radiological, or laboratory studies, as determined by the clinician. The patients who had stable disease or partial response received second-line chemotherapy instead of radiotherapy.

### Statistic analysis

Overall survival (OS) was measured from the date of diagnosis to the date of death or the last follow-up. Relapse-free survival (RFS) was calculated from the date of diagnosis to the date of disease relapse or the last follow-up. Survival functions were estimated using the Kaplan-Meier method and compared by the log-rank test. Chi-square was used for comparison of the clinical data of the patients with different treatments. Multivariate survival analysis was performed using Cox regression model. Significant variables in the univariate analysis were selected as variables in the multivariate analysis for survival. *P* < 0.05 was considered statistically significant. All statistical analyses were evaluated using Statistical Package for the Social Sciences (SPSS) 18.0 software (SPSS Inc., Chicago, IL).

## Results

### Clinical characteristics

As shown in Table 1, 64 of the 101 patients (63 %) were <=60 years old and the median age was 57 years (ranged 18 to 82 years). There were 57 male and 44 female patients. All the patients presented gastrointestinal symptoms such as abdominal discomfort (67 cases, 66 %), severe gastrointestinal bleeding (16 cases, 16 %), obstruction or intussusceptions (10 cases, 10 %), diarrhea (3 cases, 3 %), abdominal mass (6 cases, 6 %), and perforation (2 cases, 2 %).

**Table 1:** Clinicopathological characteristics of patients with localized PG-DLBCL

Characteristics		*N* (%)	5-year RFS	*P* value	5-year OS	*P* value
Age (years)	<=60	64 (63)	90.2 ± 4.2 %	0.040	91.8 ± 3.9 %	0.037
	>60	37 (37)	69.2 ± 9.3 %		67.0 ± 9.8 %	
Sex	Male	57 (56)	89.1 ± 4.6 %	0.244	90.7 ± 4.4 %	0.234
	Female	44 (44)	75.4 ± 7.6 %		74.1 ± 8.0 %	
B symptoms	No	57 (56)	79.1 ± 6.2	0.190	79.7 ± 6.5 %	0.193
	Yes	44 (44)	88.2 ± 5.6 %		87.6 ± 5.8 %	
Extranodal involvement	Single site	85 (84)	87.2 ± 4.3 %	0.040	87.8 ± 4.4 %	0.018
	Multiple sites	16 (16)	66.0 ± 12.4 %		63.6 ± 13.2 %	
LDH	Normal	77 (76)	89.0 ± 4.3 %	0.011	90.9 ± 4.0 %	0.009
	Abnormal	24 (24)	63.9 ± 11.0 %		63.6 ± 11.1 %	
β2-MG	Normal	50 (50)	94.1 ± 4.1 %	0.034	93.2 ± 4.7 %	0.025
	Abnormal	51 (50)	74.4 ± 6.7 %		75.9 ± 6.7 %	
Hypoalbuminemia	No	76 (75)	87.5 ± 4.4 %	0.036	88.3 ± 4.6 %	0.025
	Yes	25 (25)	70.3 ± 10.3 %		67.9 ± 11.0 %	
Anemia	No	56 (55)	89.3 ± 4.5 %	0.144	91.1 ± 4.3 %	0.129
	Yes	45 (45)	73.8 ± 8.1 %		71.8 ± 8.6 %	
Site of origin	Gastric	48 (47)	90.6 ± 5.2 %	0.012	92.8 ± 5.0 %	0.008
	Duodenum and small bowel	17 (17)	74.0 ± 13.2 %		70.7 ± 14.3 %	
	Ileocecal	16 (16)	86.7 ± 8.8 %		86.7 ± 8.8 %	
	Colorectal	15 (15)	84.0 ± 10.6 %		82.1 ± 11.7 %	
	Combined	5 (5)	40.0 ± 21.9 %		40.0 ± 21.9 %	

Abbreviations: *PG-DLBCL* primary gastrointestinal diffuse large B cell lymphoma, *RFS* relapse-free survival, *OS* overall survival, *LDH* lactate dehydrogenase, *β2-MG* β2-microglobulin

According to Ann Arbor staging with Musshoff modification, all the patients had localized disease (Ie to IIe_2_) with ECOG ≤2. Forty-two cases (44 %) presented with B symptoms. Multiple extranodal involvement and elevated serum LDH level were observed in 16 patients (16 %) and 24 patients (24 %), respectively. Fifty-one cases (50 %) had elevated β2-microglobulin (β2-MG), 25 cases (25 %) had hypoalbuminemia, and 45 cases (45 %) had anemia at diagnosis.

As for the sites of origin, the most frequent site was the stomach (gastric group; 48 cases, 48 %), followed by the duodenum and small bowel (17 cases, 17 %), ileocecal (16 cases, 15 %), and colorectal groups (15 cases, 15 %). The remaining 5 (5 %) patients (combined group) had both gastric and intestinal involvement.

### Surgical approaches and chemotherapy

Surgical modalities included radical surgery and alleviative surgery (74 and 27 cases, respectively). Of the 74 patients who underwent radical surgery, 9 cases received surgery alone, and the remaining 65 cases were treated with chemotherapy alone, or combined with Rituximab (21 and 44 cases, respectively). Similar distribution was found in the 27 patients who underwent alleviative surgery (3, 8, and 16 cases, respectively, Table 1).

### Pathological characteristics

Detailed pathological features of the tumors were available from operation (Table 2). Macroscopically, 12 tumors (12 %) were classified as ulcerative type, 18 (18 %) as diffuse type, and 71 (70 %) as massive type. Microscopically, the depth of invasion were limited to mucosa/submucosa (0), muscularis propria/subserosa (40, 40 %), beyond serosa (visceral peritoneum) without invasion of adjacent structures (43, 42 %), and involvement of adjacent structures or organs (18, 18 %). Involvement of regional lymph node and adjacent tissue were present in 53 and 22 patients (52 and 22 %, respectively). The high level (>75 %) of Ki-67 antigen was detected in the biopsy specimens of 25 cases (25 %). Bcl-2 expression was positive in 48 of the 101 patients (48 %).

**Table 2:** Pathological features of patients with localized PG-DLBCL

Characteristics		*N* (%)	5-year RFS	*P* value	5-year OS	*P* value
Tumor morphology	Ulcerative type	12 (12)	87.5 ± 11.7 %	0.251	87.5 ± 11.7 %	0.244
	Diffuse type	18 (18)	68.0 ± 12.0 %		66.7 ± 12.4 %	
	Massive type	71 (70)	86.9 ± 4.7 %		88.1 ± 4.6 %	
Depth of invasion	Mucosa/submucosa	0	–	0.022	–	0.029
	Muscularis propria/subserosa	40 (40)	95.8 ± 4.1 %		94.7 ± 5.1 %	
	Beyond serosa (visceral peritoneum) without invasion of adjacent structures	43 (42)	71.3 ± 8.1 %		73.7 ± 8.0 %	
	Involvement of adjacent structures or organs	18 (18)	81.1 ± 9.9 %		80.4 ± 10.2 %	
Involvement of regional lymph nodes	Negative	48 (48)	90.8 ± 5.1 %	0.038	89.4 ± 6.0 %	0.048
	Positive	53 (52)	75.8 ± 6.7 %		77.8 ± 6.5 %	
Invasion to adjacent structures or organs	No	79 (78)	87.8 ± 4.4 %	0.024	86.5 ± 4.8 %	0.028
	Yes	22 (22)	69.4 ± 10.5 %		73.6 ± 10.2 %	
High level of Ki-67	Negative	76 (75)	90.1 ± 3.8 %	<0.001	91.3 ± 3.8 %	<0.001
	Positive	25 (25)	52.6 ± 14.4 %		48.0 ± 14.5 %	
Bcl-2 expression	Negative	53 (52)	97.8 ± 2.2 %	<0.001	97.6 ± 2.4 %	<0.001
	Positive	48 (48)	65.8 ± 8.2 %		65.8 ± 8.7 %	

Abbreviations: *PG-DLBCL* primary gastrointestinal diffuse large B cell lymphoma, *RFS* relapse-free survival, *OS* overall survival

### Treatment outcome

The overall CR, PR, and SD/PD rate were 73, 15, and 12 %, respectively. The median follow-up time was 23 months (ranged 1 to 115 months). Overall, the 5-year RFS and OS rates were 82.5 ± 4.5 % and 83.9 ± 4.4 %, with median RFS and OS at 43.3 and 49.6 months, respectively.

By univariate analysis, the clinical characteristics significantly correlated with poor RFS and OS in the patients who received surgery were age older than 60 years old, the presence of multiple extranodal involvement, elevated serum LDH level and β2-MG, and decreased serum albumin (Table 1). Regarding pathological parameters, adverse prognostic factors included invasion depth beyond serosa, involvement of regional lymph nodes or adjacent tissue, high level of Ki-67, and Bcl-2 expression (Table 2).

Gastric, duodenum and small bowel, ileocecal, and colorectal group showed a higher survival rate than those with multiple sites involved (Table 1). To determine the role of surgery in the treatment of localized PG-DLBCL, we included 49 patients who received chemotherapy alone as control. As showed in Additional file 1: Table S1, no significant difference of clinical characteristics and Rituximab treatment was observed between the patients with surgery or those with chemotherapy alone. Surgery did not prolong the survival rate of localized PG-DLBCL patients, when compared with chemotherapy alone. However, in the patients without Rituximab treatment, mostly due to active infection of hepatitis B virus, the survival rate showed longer RFS and OS in cases who received radical surgery than those with alleviative surgery (RFS, 88.5 ± 4.4 % vs 69.5 ± 9.7 %; OS, 87.7 ± 4.8 % vs 72.3 ± 9.7 %, both *P* = 0.004). Addition of Rituximab significantly improved the survival of the patients who received alleviative surgery and chemotherapy (RFS, 92.9 ± 6.9 % vs 58.3 ± 18.6 %, *P* = 0.002; OS, 91.7 ± 8.0 % vs 71.4 ± 17.1 %, *P* = 0.001), instead of those who received radical surgery and chemotherapy (RFS, 93.8 ± 4.3 % vs 88.2 ± 7.8 %, *P* = 0.302; OS, 93.3 ± 4.6 % vs 88.2 ± 7.8 %, *P* = 0.333, Table 3).

**Table 3:** Treatment modalities of patients with localized PG-DLBCL

Treatment	*N*	5-year RFS	*P* value	5-year OS	*P* value
Radical surgery	74	88.5 ± 4.4 %	0.004	87.7 ± 4.8 %	0.004
Alleviate surgery	27	69.5 ± 9.7 %		72.3 ± 9.7 %	
Radical surgery and chemotherapy with Rituximab	44	93.8 ± 4.3 %	0.302	93.3 ± 4.6 %	0.333
Radical surgery and chemotherapy without Rituximab	21	88.2 ± 7.8 %		88.2 ± 7.8 %	
Alleviate surgery and chemotherapy with Rituximab	16	92.9 ± 6.9 %	0.002	91.7 ± 8.0 %	0.001
Alleviate surgery and chemotherapy without Rituximab	8	58.3 ± 18.6 %		71.4 ± 17.1 %	

Abbreviations: *PG-DLBCL* primary gastrointestinal diffuse large B cell lymphoma, *RFS* relapse-free survival, *OS* overall survival

By multivariate analysis, the significant independent prognostic factors for poor RFS and OS was multiple gastrointestinal involvement.

### Staging systems

**Fig. 1:**
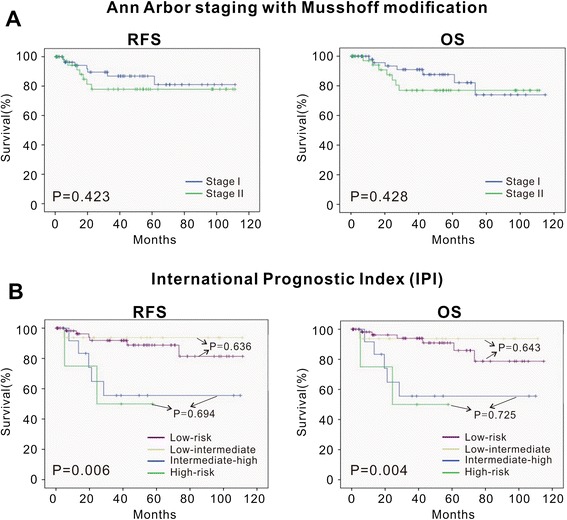
The RFS and OS curve according to Ann Arbor stage modified by Musshoff (**a**) and IPI score (**b**). The relapse-free survival (RFS) and overall survival (OS) curves according to Ann Arbor stage modified by Musshoff (**a**) and IPI score (**b**) show that these staging systems could define specific risk subgroups of patients with localized PG-DLBCL to some extent

**Fig. 2:**
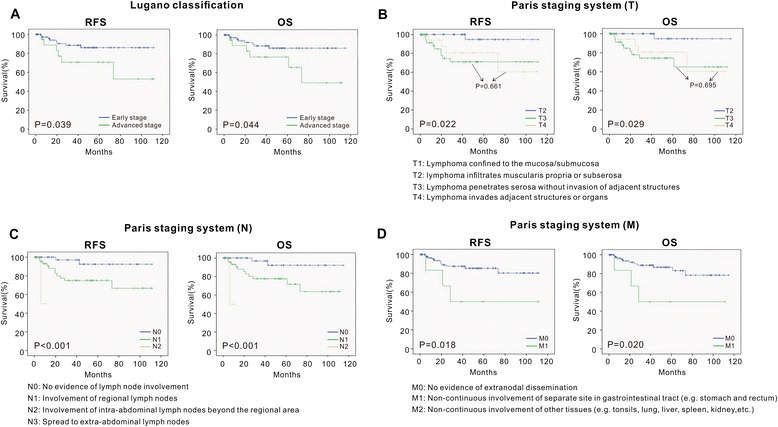
The RFS and OS curves according to Lugano classification (**a**) and Paris staging system (**b**–**d**). The relapse-free survival (RFS) and overall survival (OS) curves according to Lugano classification (**a**) and Paris staging system (**b**–**d**) show that these staging systems could define specific risk subgroups of patients with localized PG-DLBCL to some extent

**Fig. 3:**
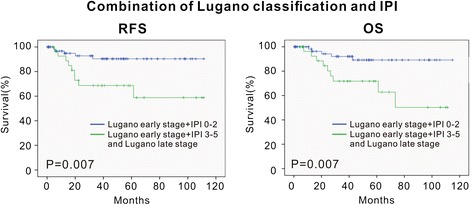
The RFS and OS curves according to the combination of Lugano classification and IPI. The relapse-free survival (RFS) and overall survival (OS) curves according to combination of Lugano classification and IPI shows that the combination of clinical and pathological staging system was more efficient in classifying PG-DLBCL patients

As illustrated in Figs. 1 and 2, the staging systems varied from each other for defining specific risk subgroups. Ann Arbor staging with Musshoff modification could not further stratify the early-stage patients into different stages (stage I and stage II) (I, 5-year RFS, 86.8 ± 5.1 %; 5-year OS, 87.6 ± 5.3 % vs II, 5-year RFS, 77.9 ± 7.4 %; 5-year OS, 76.9 % ± 7.7 %, *P* = 0.423 and *P* = 0.428, respectively). IPI was able to define specific risk subgroups (low/low–intermediate (L–I)-risk and intermediate–high (I–H)/high-risk), but there was no prognostic difference between the low-risk subgroup and the L–I-risk group (5-year RFS and 5-year OS, *P* = 0.636 and *P* = 0.643, respectively), or between the high–intermediate (H–I)-risk subgroup and the high-risk group (5-year RFS and 5-year OS, *P* = 0.694 and *P* = 0.725, respectively). Using Lugano classification, the patients with advanced stage (IIE) had significantly shorter survival time than those with early stage (I and II) (IIE, 5-year RFS, 71.1 ± 11.0 %; 5-year OS, 76.0 ± 10.5 % vs I and II, 5-year RFS, 86.5 ± 4.5 %; 5-year OS, 85.4 ± 4.9 %, *P* = 0.039 and *P* = 0.044, respectively). Using Paris staging system, the patients in T3 and T4 showed no significant survival difference (T3, 5-year RFS, 71.3 ± 8.1 %; 5-year OS, 73.7 ± 8.0 % vs T4, 5-year RFS, 81.1 ± 9.9 %; 5-year OS, 80.4 ± 10.2 %, *P* = 0.661 and *P* = 0.695, respectively) (Table 4). Of note, according to IPI, Lugano early stage was grouped to IPI 0–2 (72 patients) and IPI 3–5 (10 patients) (Fig. 3). The latter had similar RFS and OS of the cases with Lugano late stage (*P* = 0.960 and *P* = 0.870, respectively). Thus, combination of clinical and pathological staging system was more efficient in classifying PG-DLBCL patients.

**Table 4:** Staging systems of patients with localized PG-DLBCL

Staging system	Stage		*N* (%)	5-year RFS	*P* value	5-year OS	*P* value
Ann Arbor staging with Musshoff modification	I (Ie_1_–Ie_2_)		62 (61)	86.8 ± 5.1 %	0.423	87.6 ± 5.3 %	0.428
	II (IIe_1_–IIe_2_)		39 (52)	77.9 ± 7.4 %		76.1 ± 7.7 %	
IPI	Low		65 (64)	89.4 ± 4.5 %	0.006	90.9 ± 4.4 %	0.004
	L–I		19 (19)	93.8 ± 6.1 %		93.8 ± 6.1 %	
	H–I		4 (4)	56.3 ± 14.8 %		55.6 ± 14.9 %	
	High		13 (13)	50.0 ± 2.5 %		50.0 ± 2.5 %	
Lugano classification	Early stage (I–II)		82 (81)	86.5 ± 4.5 %	0.039	85.4 ± 4.9 %	0.044
	Late stage (IIE)		19 (19)	71.1 ± 11.0 %		76.0 ± 10.5 %	
Paris staging system	T	1	0	–	0.022	–	0.029
		2	40 (40)	95.8 ± 4.1 %		94.7 ± 5.1 %	
		3	43 (42)	71.3 ± 8.1 %		73.7 ± 8.0 %	
		4	18 (18)	81.1 ± 9.9 %		80.4 ± 10.2 %	
	N	0	48 (48)	93.5 ± 4.5 %	<0.001	92.2 ± 5.4 %	<0.001
		1	51 (50)	75.0 ± 6.9 %		77.2 ± 6.7 %	
		2	2 (2)	50.0 ± 35.4 %		50.0 ± 35.4 %	
		3	0	–		–	
	M	0	95 (94)	85.9 ± 4.2 %	0.018	86.2 ± 4.3 %	0.020
		1	6 (6)	50.0 ± 20.4 %		50.0 ± 20.4 %	
		2	0	–		–	

Abbreviations: *PG-DLBCL* primary gastrointestinal diffuse large B cell lymphoma, *RFS* relapse-free survival, *OS* overall survival, *IPI* International Prognostic Index, *L–I* low–intermediate, *H–I* high–intermediate

## Discussion

PG-DLBCL represents the most common subtype of extranodal lymphoma, mainly involved in stomach and bowl [19]. Comparable to previous studies in Western and Asian countries [20–26], clinical parameters associated with deteriorated patient status (older age and hypoalbuminemia) as well as increased tumor burden (multiple extranodal and gastrointestinal involvement, elevated LDH, and β2-MG) were important factors indicating poor prognosis. Among all these univariate prognostic factors, multiple gastrointestinal involvement was independently related to adverse outcome of the patients. With the development of endosonography, radiological examination and PET-CT, patients with multiple gastrointestinal involvement could be easily distinguished nowadays. Also, pathological parameters negatively correlated with disease prognosis were identified, including tumor infiltration and involvement of regional lymph nodes, adjacent structures or organs, high Ki-67, and Bcl-2 expression. As previously reported, Ki-67 reflects high proliferation index and Bcl-2 is an important anti-apoptotic protein [27, 28], both of which correlate with the aggressive course in patients with DLBCL. Therefore, in addition to clinical prognosticators, pathological characteristics that are associated with biological behavior of the tumors are meaningful for appropriate prognostic settings of the patients with PG-DLBCL.

Rituximab, a chimeric anti-CD20 antibody, is generally applied to treat B cell lymphoma. Like nodal lymphomas, the survival of primary gastric B cell lymphoma has been improved upon Rituximab treatment [29, 30]. Interestingly, the negative impact of alleviate surgery could be overcome by Rituximab treatment. Meanwhile, based on our data and the others [24, 31, 32], radical surgery may be considered as a therapeutic modality to patients unfit for Rituximab treatment (active infection of hepatitis B virus, etc.).

Staging systems are important to provide adequate treatment guidance. For the early stage of localized PG-DLBCL patients, Ann Arbor staging with Musshoff modification failed to give prognostic indications. Instead, IPI appeared more efficient in dividing the patients into two risk subgroups with distinct outcome, but it was unable to separate the survival of patients with low and L–I risks, as well as high and H–I risks [28, 33, 34]. Lugano classification [12, 25], including major pathological parameters like the depth of infiltration and infiltration of adjacent organs, also proved efficient. Interestingly, our data showed that IPI in conjunction with Lugano classification could further improve their capacity to discriminate the important risk subgroups. Therefore, the combination of clinical and pathological staging systems is optimal to predict the prognosis of PG-DLBCL.

## Conclusions

Non-surgical treatment becomes an optimal therapeutic modality for localized PG-DLBCL in the Rituximab era. Addition of Rituximab might overcome the negative prognostic effect of alleviative surgery. The combination of pathological staging system and clinical system is optimal for prognosis prediction in patients with PG-DLBCL.

## Additional file

Additional file 1: Table S1. Clinical characteristics and survival rate of patients with localized PG-DLBCL. It shows the clinical characteristics and Rituximab treatment between localized PG-DLBCL patients with surgery and those with chemotherapy alone. (PDF 276 kb)

## Abbreviations

CR: complete response
DLBCL: diffuse large B cell lymphoma
IPI: International Prognostic Index
LDH: lactate dehydrogenase
OS: overall survival
PD: progression disease
PG-DLBCL: primary gastrointestinal DLBCL
PR: partial response
RFS: relapse-free survival
SD: stable disease
WHO: World Health Organization
β2-MG: β2-microglobulin
