# Supplementary material for: Localized primary gastrointestinal diffuse large B cell lymphoma received a surgical approach: an analysis of prognostic factors and comparison of staging systems in 101 patients from a single institution
Source: World J Surg Oncol. 2015 Aug 15;13:246. doi: 10.1186/s12957-015-0668-5 (PMC4536702; doi:10.1186/s12957-015-0668-5)
Supplement: Additional file 1: Table S1. — Clinical characteristics and survival rate of patients with localized PG-DLBCL. It shows the clinical characteristics and Rituximab treatment between localized PG-DLBCL patients with surgery and those with chemotherapy alone. (PDF 276 kb) [file 12957_2015_668_MOESM1_ESM.pdf]

Additional file 1: Table S1 Clinical characteristics and survival rate of patients with localized PG-DLBCL

	**Surgery (n=101)**	**Chemotherapy (n=49)**	**P value**
**Age >60 yrs (%)**	40 (39.6%)	17 (34.7%)	0.561
**IPI 3-5 (%)**	17 (16.8%)	7 (14.3%)	0.690
**Abnormal LDH (%)**	24 (23.8%)	13 (26.5%)	0.712
**Abnormal β2-MG (%)**	51 (50.5%)	30 (61.2%)	0.216
**Rituximab treatment (%)**	60 (59.4%)	37 (75.5%)	0.053
**5-yr RFS**	83.1%±4.3%	71.6%±9.2%	0.260
**5-yr OS**	83.3%±4.5%	71.1%±9.6%	0.524

Abbreviations:

PG-DLBCL: primary gastrointestinal diffuse large B-cell lymphoma; IPI: International Prognostic Index; LDH: lactate dehydrogenase; β2-MG: β2-microglobulin; RFS: relapse-free survival; OS: overall survival
